# Gender inequality in source country modifies sex differences in stroke incidence in Canadian immigrants

**DOI:** 10.1038/s41598-022-22771-3

**Published:** 2022-10-26

**Authors:** Manav V. Vyas, Jiming Fang, Moira K. Kapral, Amy Y. X. Yu, Peter C. Austin

**Affiliations:** 1 Division of Neurology, Department of Medicine, University of Toronto, Toronto, Canada; 2 ICES, Toronto, Canada; 3 Institute of Health Policy, Management and Evaluation, University of Toronto, Toronto, Canada; 4 Division of General Internal Medicine, Department of Medicine, University of Toronto, Toronto, Canada; 5 St. Michael’s Hospital-Unity Health Toronto, 6th Floor Bond Wing (Neurology Clinic), 30 Bond Street, Toronto, ON M5B 1W8 Canada

**Keywords:** Stroke, Risk factors, Epidemiology

## Abstract

Research suggests that gender inequality, measured using the gender inequality index (GII), influences stroke mortality in women compared to men. We examine how source country GII modifies the rate of ischemic stroke in women compared to men after immigration to Canada, a country with low gender inequality. We used linked health data and immigration records of 452,089, stroke-free immigrants aged 40–69 year who migrated from 123 countries. Over 15 years of follow-up, 5991 (1.3%) had an incident ischemic stroke. We demonstrate (a) a lower adjusted rate of stroke in women compared to men (hazard ratio 0.64; 95% CI 0.61–0.67); (b) that sex differences in stroke incidence were modified by source country GII, as the hazard of stroke in women vs. men attenuated by a factor of 1.06 for every 0.1 increase in the GII of the source country (P_sex*GII_ = 0.002); and (c) migration to a country with low GII attenuates the adverse effect of source country GII on sex differences in stroke incidence. Evaluating pathways through which source country gender inequality differentially influences stroke risk in immigrant women compared to men could help develop strategies to mitigate the effects of early-life gender inequality on stroke risk.

## Introduction

Early life adversity is being increasingly recognized as an independent risk factor for poor cardiovascular health in adult life^1^. Gender inequalities early in life, measured using four domains of health, education and transition to employment, protection, and a safe environment, are associated with considerable disadvantages beyond childhood^2^. A commonly used measure of gender inequality is the gender inequality index (GII) which is a measure of potential human development loss due to inequality between women and men based on reproductive health, education, and economic status as defined by the United National High Commissioner for Refugees (UNHCR)^3^. It is calculated using the association-sensitive inequality method suggested by Seth, and weights health, education and economic status equally^4^. It ranges between 0 and 1, with higher values indicating greater inequality between women and men. Ecological studies have found lower participation in physical activities^5^, and higher rates of obesity^6^ in women compared to men in countries with higher GII compared to those with lower GII, but they did not account for individual-level risk factors. Further, the modifying effects of the components of GII, education and income, on sex differences in cardiovascular disease have been reported^7^. Yet, the modifying role of early life gender inequality on the sex differences in the incidence of cardiovascular disease is not well-studied, especially in people who immigrate to a country with low GII. We focus our attention on ischemic stroke because it is the most common stroke type and a leading cause of disability worldwide^8^.

Canada is among the top 20 countries with low gender inequality (GII = 0.08), and up to one-fifth (21% in 2016) of its total population consists of immigrants who have arrived from countries across the world with varying early life experiences^9^. Sex differences in the incidence of ischemic stroke are well-studied, with prior work suggesting a lower rate of ischemic stroke in women compared to men among adults aged 40 years and over^10,11^. Because gender inequality among Canadian non-immigrants is likely to be low and homogenous, understanding sex differences in stroke incidence among Canadian immigrants, and examining whether this association varies by source country GII can provide a unique opportunity to evaluate the effect of early life gender inequality on the differential risk of stroke in women compared to men.

Our objective was to evaluate if gender inequality, measured as GII of the source country (i.e., country of citizenship at the time of immigration), modifies the rate of ischemic stroke in women compared to men among adult Canadian immigrants. We hypothesized that a higher source country GII would be associated with an increased rate of stroke in immigrant women compared to immigrant men.

## Methods

In Ontario, the provincial health insurance plan covers costs associated with medically necessary healthcare services, including hospitalizations, outpatient, and emergency visits. All immigrants, except transient visitors and undocumented migrants, are covered under this provincial health insurance plan, and this allows us to identify all index stroke events. We conducted a retrospective cohort study of community-dwelling immigrants residing in Ontario, Canada, aged 40 to 69 years on January 1, 2003 (cohort inception), who were eligible for the provincial health insurance plan one year prior to cohort inception, and had no prior history of stroke or TIA^12^ (look back to 1990). We restricted the sample to those aged 40 to 69 years because of the known sex-specific variations in stroke risk at younger and older age groups^11^. We excluded people who were born in Canada, and those born outside of Canada but arrived in Canada before 1985 as our immigration dataset, obtained from the Immigration, Refugees and Citizenship Canada Permanent Resident Database, only provides information from 1985 onward. We further excluded immigrants from countries for which the total number of eligible cohort participants was less than 30. Using unique identifiers, we linked the data of this cohort to administrative databases, including postal code files, census files, hospitalizations, and emergency visit databases.

### Exposures and outcomes

Our primary exposures were sex and source country GII. Sex was determined based on the registered persons database which includes sex at the time of application to the provincial health plan. We ascertained GII of the source country based on the UNHCR database. GII is a measure of position of women in a country, with higher values suggesting greater gender inequality, and this was available for most countries for the year 2005, the year closest to cohort inception.

Our primary outcome was occurrence of first-ever ischemic stroke. We defined this as an emergency department visit or acute care hospitalization with a diagnosis of ischemic stroke, using International Statistical Classification of Diseases and Related Health Problems (ICD)-10 codes: H34.1, I63.x, I64.x^13^. If an individual had more than one event during follow-up, we included the first event in the primary analyses. We set the end date of follow-up as March 31, 2018.

### Statistical analyses

We reported standardized differences to compare the baseline characteristics between immigrant women and men, with standardized differences greater than 0.10 considered reflective of a potentially meaningful difference^14^.

We used a series of three cause-specific hazard regression models. In the first model, we regressed the hazard of stroke on sex using a cause-specific hazard model to account for the competing risk of death and that of loss to follow-up (model #1). This model was used to estimate the overall hazard ratio (HR) of stroke in women compared to men, adjusted for age, neighbourhood-level income (based on 2011 Census data), hypertension, diabetes, dyslipidemia, atrial fibrillation, congestive heart failure and chronic obstructive pulmonary disease, and immigration-related factors such as immigration class and proportion of life spent in Canada (e-Table 2 has details on validated algorithms used to derive these variables). In the second model, we used frailty (or random effects) cause-specific hazards models, incorporating country-level random effects, to estimate the unadjusted hazard ratio (HR) of ischemic stroke in women compared to men (model #2). This model incorporated random slopes for sex, allowing the association between sex and the log-hazard of stroke to vary across countries^15^. Based on this second model (model #2), which only included country-specific sex effects (and no other covariates), we plotted the unadjusted predicted HR of stroke in women vs. men against the GII for each included country. We then used the ordinary least squares (OLS) models to depict the unadjusted linear association between GII and predicted country-level HR of stroke in women vs. men and reported the magnitude and strength using point estimate for the slope and the P values. Lastly, in the third model, to evaluate the modifying role of GII on the HR of stroke in women vs. men, we fitted a multivariable cause-specific random effects model that included a cross-level interaction between country-level GII and sex (model #3). We reported the beta-coefficient and P value of the interaction term.

### Secondary analyses

Immigrants to Canada can migrate based on three major categories: economic, family and refugee. Economic immigrants migrate based on points earned for education, employment, language skills and age; family class immigrants migrate to unite with their families already residing in Canada; and refugees in Canada are either seeking asylum through private or government sponsorship, or because of risk of persecution or danger in their home countries. For economic migration for families using the points-based system, the principal applicant’s education, employment, and language skills contribute to the bulk of the overall points whereas their spouse or partner’s education, employment and language skills have a smaller contribution^16^. Recognizing that the GII of countries from where immigrants arrive as refugees (e.g., Afghanistan) would be higher compared to GII of countries from where immigrants arrive as economic migrants (e.g., United Kingdom), we performed subgroup analyses stratified by immigration class using unadjusted and adjusted cause-specific random effects models as above.

### Comparing to the Global Burden of Disease Study

We compared our associations between source country GII and the rate of ischemic stroke in women vs. men among Canadian immigrants to that obtained from the Global Burden of Disease Study (GBD) (available from http://ghdx.healthdata.org/gbd-results-tool), which is available for use through Open Data Commons Attribution License (non-commercial users). We plotted the age-adjusted rate ratio of incident ischemic stroke in women compared to men in the included countries against the GII of these countries using GBD data and created a similar graph using hierarchical Poisson models (to obtain rate ratios) in our sample of Canadian immigrants. We then used the ordinary least squares (OLS) models to depict the unadjusted linear association between GII and rate ratios of stroke in women vs. men in both samples and reported the magnitude and strength using point estimate for the slope and the P values.

Use of the data in this study was authorized under section 45 of Ontario’s Personal Health Information Protection Act, which does not require review by a Research Ethics Board or consent from the included participants.

## Results

We followed 452,089 adult Canadian immigrants, of whom 49.5% were women, for a median time of 15 years from January 1, 2003 to March 31, 2018 (e-Fig. 1). Women were marginally older at cohort inception than men [mean age of women 49.6 years [(standard deviation (SD) 7.7 years) vs. men 48.7 (SD 7.4), standardized difference 0.13]. Compared to men, women were less likely to arrive as refugees (11.4% vs. 17.9%, standardized difference 0.19) and more likely to arrive as family class immigrants (39.7% vs. 28.9%, standardized difference 0.23). Other baseline characteristics are shown in Table 1.

**Table 1:** Characteristics of adult immigrants (aged 40–69 years) residing in Ontario, Canada as of cohort start date, January 1, 2003 (N = 452,089).

Characteristic	Women n = 223,600 (49.5%)	Men n = 228,489 (50.5%)	Standardized difference
Mean age at cohort start	49.6 (8.1)	48.7 (7.4)	0.13
Mean gender inequality index (GII) of country of origin	0.38 (0.17)	0.39 (0.18)	0.03
Age of arrival to Canada	40.4 (9.20)	39.4 (8.58)	0.12
Number of years living in Ontario	9.2 (4.40)	9.3 (4.51)	0.03
Proportion of life in Ontario	0.19 (0.10)	0.20 (0.09)	0.05
**Immigration class, n (%)**			
Economic	109,292 (48.9)	121,443 (53.2)	0.09
Family	88,849 (39.7)	66,054 (28.9)	0.23
Refugee	25,459 (11.4)	40,992 (17.9)	0.19
**Neighbourhood-level income**			
Lowest quintile	72,615 (32.5)	74,435 (32.6)	0.00
Highest quintile	25,621 (11.5)	25,122 (11.0)	0.02
**Comorbidities**			
Hypertension	45,633 (20.4)	40,931 (17.9)	0.06
Diabetes	18,419 (8.2)	20,585 (9.0)	0.03
Congestive heart failure	1132 (0.5)	1195 (0.5)	0.00
Dyslipidemia	1226 (0.5)	2678 (1.2)	0.07
Atrial fibrillation	568 (0.3)	851 (0.4)	0.02
Chronic obstructive pulmonary disease	6101 (2.7)	7856 (3.4)	0.04

**Figure 1:**
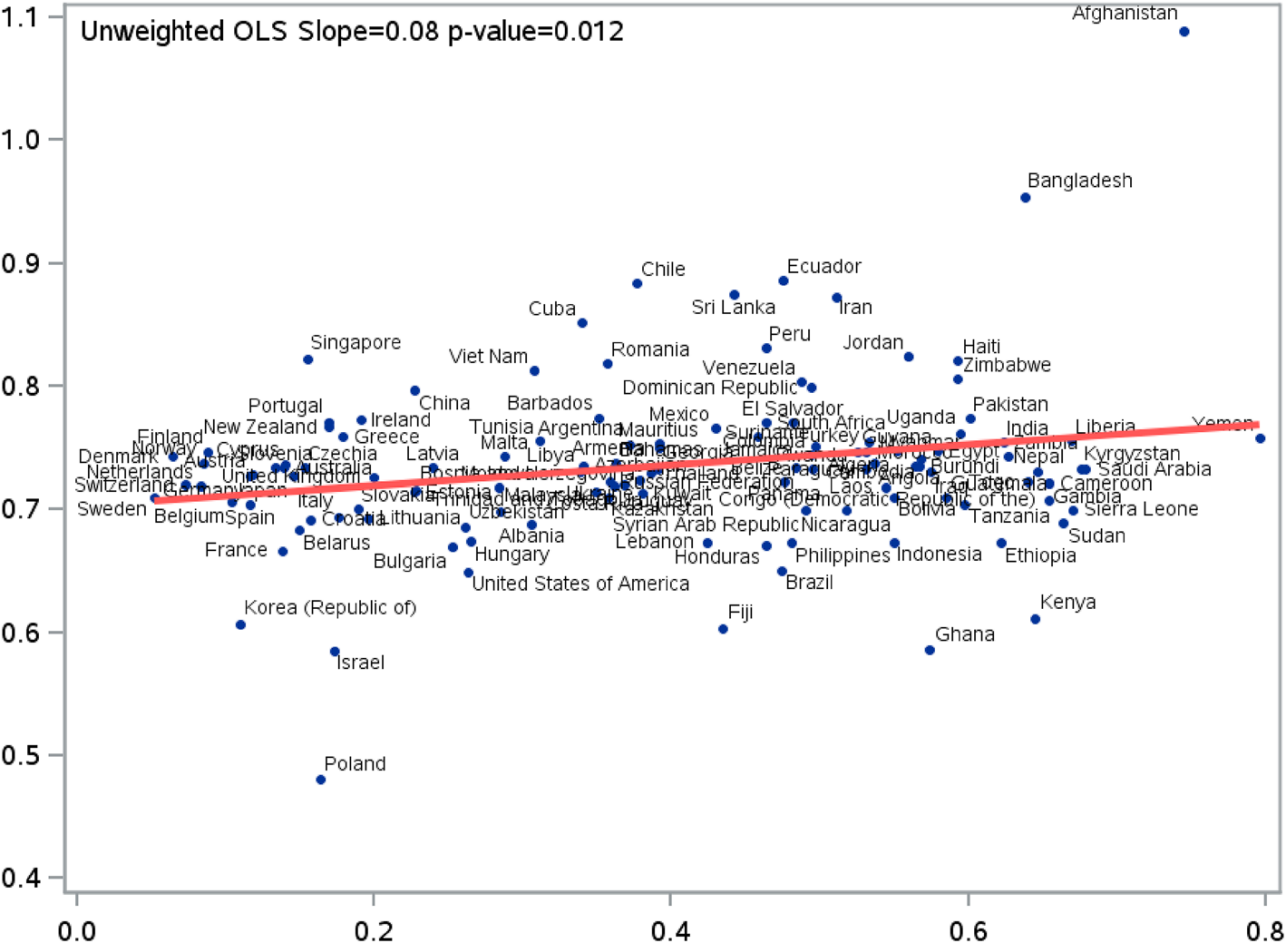
Results of hierarchical model-specific predicted country-specific hazard ratios (HR) of ischemic stroke in women compared men (Y-axis) and their association with country-level gender inequality index (GII) (X-axis) presented using unweighted linear regression. Predicted country-specific HRs are obtained from a model that adjusts only for sex and allows random slopes for sex.

During 6.2 million person-years follow-up, 5991 (1.3%) individuals of the total study sample had an incident ischemic stroke. The adjusted hazard of ischemic stroke was lower in women compared to men (HR 0.64, 95% confidence interval 0.61–0.67) (Table 2). We present unadjusted linear association between source country GII and the random effects model (model #2)-based predicted country-specific HR of ischemic stroke in women vs. men in Fig. 1. Based on the linear model, greater GII was associated with attenuation of the lower hazard of stroke in women vs. men (OLS slope = 0.08, and P = 0.012).

**Table 2:** Results of multivariable Cox-proportional hazard models evaluating the association between sex and incidence of ischemic stroke in adult immigrants (aged 40–69 years) residing in Ontario, Canada (N = 452,089).

Characteristics	Hazard ratio (95% CI)	P value
Women vs. men	0.64 (0.61–0.67)	< 0.0001
**Neighbourhood-level income quintile**		
First (lowest)	1.50 (1.35–1.66)	< 0.0001
Second	1.39 (1.25–1.55)	< 0.0001
Third	1.35 (1.21–1.51)	< 0.0001
Fourth	1.21 (1.07–1.36)	0.002
Fifth (highest)	Reference	
**Comorbidities**		
Hypertension	1.96 (1.85–2.07)	< 0.0001
Diabetes	1.89 (1.78–2.01)	< 0.0001
Hyperlipidemia	1.33 (1.16–1.54)	< 0.0001
Congestive heart failure	1.70 (1.43–2.01)	< 0.0001
Atrial fibrillation	1.72 (1.40–2.12)	< 0.0001
Chronic obstructive pulmonary disease	1.11 (0.99–1.24)	0.08
**Immigration class**		
Family or other	1.28 (1.21–1.37)	< 0.0001
Refugee	1.39 (1.29–1.50)	< 0.0001
Economic	Reference	
Proportion of life spent in Canada (for every 10% increase)	1.03 (1.00–1.07)	0.03

*CI* confidence interval.

In the adjusted hierarchical random effects model, source country GII modified the association between the HR of stroke in women compared to men ($\beta$_sex*GII_ = 0.56; P_sex*GII_ = 0.002) such that a 0.1 increase in values of GII attenuated the lower rate of stroke in women compared to men by 1.06 (95% CI 1.02–1.10) (e-Table S1). When we performed *ad-hoc* sensitivity analyses removing countries with very high (Afghanistan and Bangladesh) and very low GII (Poland), we found that association between country-level GII and hazard of stroke in women compared to men was attenuated (OLS slope 0.002, P = 0.12), and the sex × GII interaction was no longer significant in hierarchical models ($\beta$_sex*GII_ = 0.18, P_sex*GII_ = 0.33).

**Figure 2:**
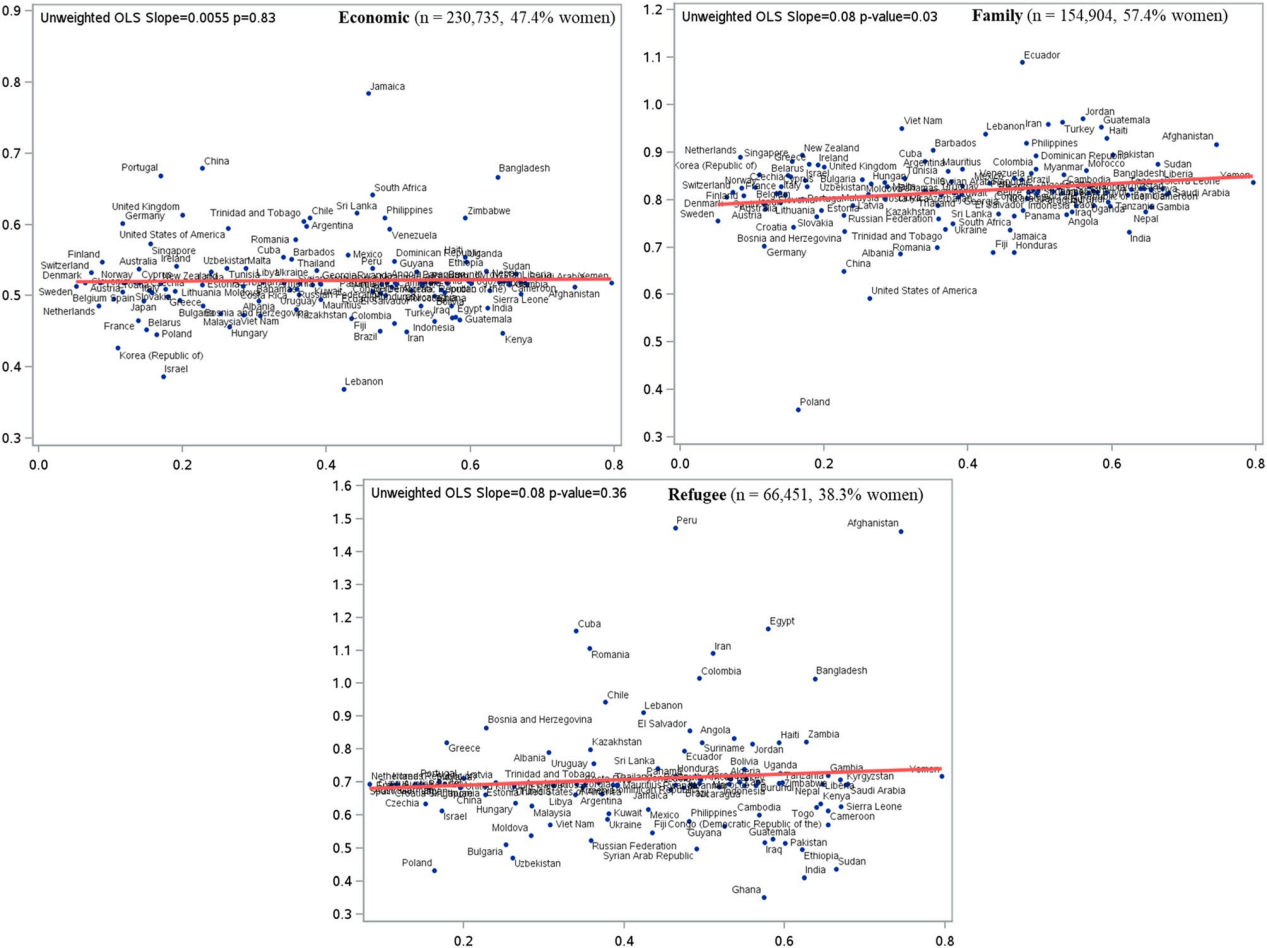
Results of hierarchical model-specific predicted country-specific hazard ratios (HR) of ischemic stroke in women compared men (Y-axis) and their association with country-level gender inequality index, GII, (X-axis) stratified by immigration class.

In subgroup analyses by immigration class, in both unadjusted (Fig. 2) and multivariable adjusted hierarchical models, the modifying effect of source country GII on HR of stroke in women compared to men was only noted in family class migrants ($\beta$_sex*__GII_ = 0.79; P_sex*GII_  = 0.005), but not refugees ($\beta$_sex*__GII_ = 0.26; P_sex*GII_ = 0.74) or economic migrants ($\beta$_sex*_ _GII_ = 0.12; P_sex*GII_ = 0.71).

### Comparing to the Global Burden to Disease Study

**Figure 3:**
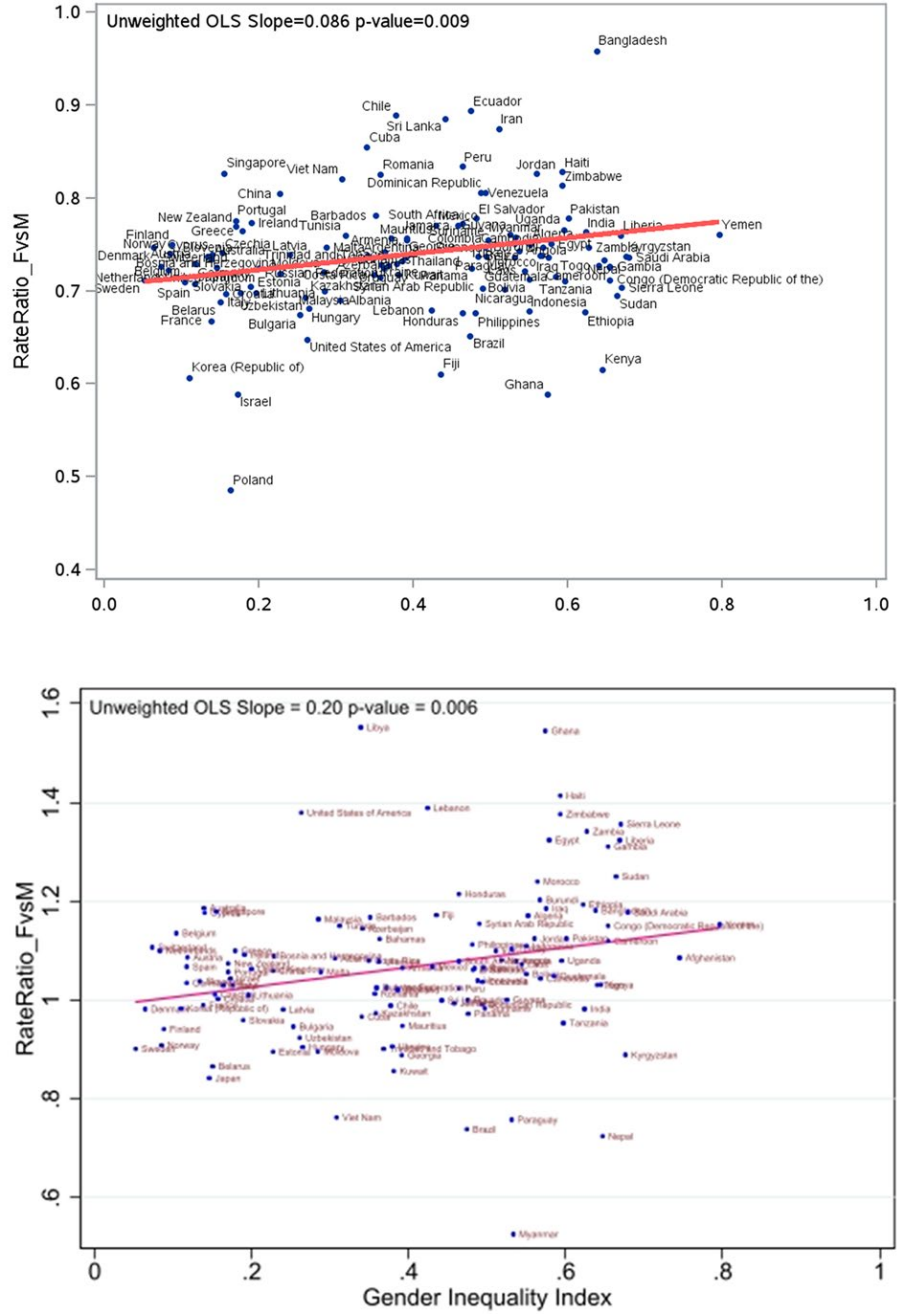
Comparing the relationship between country-level GII and predicted rate ratio of ischemic stroke in women compared to men (F vs. M) among Canadian immigrants (top panel) and observed rate ratios of ischemic stroke (F vs. M) among people in their home countries (bottom panel) based on Global Burden of Disease data.

Figure 3 compares the association between GII and the rate ratio of stroke in women vs. men in Canadian immigrants and the GBD sample. The unadjusted association between country-level GII and the rate ratio of stroke in women compared to men, measured as the slope of linear regression, was significant in both the GBD sample (OLS slope = 0.2; P = 0.006) and Canadian immigrants (OLS slope = 0.09, P = 0.009).

## Discussion

Among 400,000 middle-aged Canadian immigrants who arrived in Canada in adulthood, we found an overall lower hazard of ischemic stroke in women compared to men, consistent with other studies^10,11^. However, the lower hazard in women vs. men attenuated as the GII in the source countries increased: the higher the gender inequality of the source country, the greater the attenuation of the lower stroke risk in women compared to men. The modifying effect of source country GII on the sex differences in incidence of ischemic stroke was significant among family class immigrants, but not refugees or economic immigrants.

A higher risk of poor mental health and assault among Canadian immigrant women who arrived from countries with higher GII values than those who arrived from countries with lower GII has been previously reported^17^, but the effect of source country GII on other health conditions, especially cardiovascular disease, has not been previously studied. Not only did we demonstrate an association between the source country GII on the hazard of stroke in women compared to men in non-hierarchical models, but by using hierarchical models, we showed that source country GII modifies the association between sex and hazard of ischemic of stroke, a significant interaction effect, despite adjusting for individual-level risk factors. While the ad-hoc sensitivity analyses showed null effects, we exercise caution in concluding null effects because the observed very high or low GII and responding predicted HR for the three countries (Afghanistan, Bangladesh, and Poland) are due true variation, and not a measurement error. Our findings are similar to previous work using GBD data that found an association between country-level GII and stroke mortality in women^18^. However, our work extends previous work by evaluating the outcome of stroke incidence at an individual rather than country level, potentially reducing the ecological fallacy^19^. Further, by comparing the association between GII and rate ratio of ischemic stroke in the GBD data and among immigrants to Canada, we demonstrate that (a) the risk of stroke in women compared to men is lower among Canadian immigrants whereas it is similar in women and men in the GBD data, and (b) the influence of gender inequality on sex differences in stroke risk could be marginally attenuated by migration to a country with low GII even in later life. It is important to note that the GBD data is derived from both immigrants and non-immigrants in the respective countries, and the healthy immigrant effect and the GII effect are at play in the Canadian immigrants cohort, suggesting cautious interpretation when directly comparing these two datasets.

Gender norms are socially or culturally constructed. These norms have been shown to determine roles and opportunities and thus determine health, health risk behaviours, and access to quality of health services^20^. Harmful or restrictive gender norms, values, and expectations (i.e., gender inequality) can be present in early life, resulting in health inequalities that can extend across the life course^21^. The pathways through which early life gender inequality modifies risk of stroke in adulthood are not entirely known. That GII modified sex differences in ischemic stroke among family class immigrants, but not economic migrants who are generally more educated and possess wealth/savings to be able to migrate^9^, could suggest that education and economic position could be important pathways through which early life gender inequality influences adult health. Previous work on early life adversity and cardiovascular disease in adulthood in the United States (US) suggests poor educational attainment, poor health behaviours and financial stress as potential pathways^22^. Among first generation immigrants in the US, over half of the overall variation in gender gap in labour force participation could be explained by gender gap in home country labour force participation rates^23^. Yet, in the European Union where the educational and financial systems are relatively homogenous, higher gender inequality was associated with a larger gender gap in life expectancy^24^, suggesting alternate pathways through which gender inequality may influence stroke risk and cardiovascular disease.

Certain limitations merit discussion. GII is measured at a country-level and being an immigrant from a country with high gender inequality may not confer individual-level gender inequality. Further, source country GII was obtained from a single year (2005), and may have changed with time^3^. We could not account for lifestyle risk factors such as smoking, alcohol use and sedentary behaviour, or know the health status of immigrants prior to immigration to Canada, all of which could influence the risk of ischemic stroke and can vary based on country of origin and sex^25^. Immigrants in our cohort arrived in Canada as adults, and we a priori excluded people from those countries that had smaller contributions (less than 30 participants) to the total number of immigrants in Canada. Thus, our findings may only generalize to certain immigrant populations. It would be valuable to study the influence of source country GII on sex differences in health outcomes among immigrants who arrived as children (less than 5 or 10 years) compared to those who arrived as adults, when the gender norms are well-developed. We could not distinguish economic immigrants as principal applicant or not, but recognize that there may be variation in education, employment and language skills amongst members of a family immigrating together under the economic class. In addition, longitudinal measurement of covariates to allow for variation over time of factors such as neighbourhood-level income and vascular risk factors could be useful in future studies. Sex was a biological measure in our analyses as we could not ascertain self-identified gender in administrative data. Future work should incorporate self-identified gender, including non-binary roles, and identify individual-level measures of gender inequality, such as education attainment, employment, or participation in sports to fully understand the role of gender inequality on sex differences in stroke risk^26^. Lastly, longitudinal ascertainment of GII at time of immigration may be useful to account for changes in source country GII over time.

In summary, our findings support the concept that early-life gender inequality can differentially influence the rate of stroke in women compared to men, even among immigrants to a country with low gender inequality. We need to further understand how gender norms formed early in life evolve over the life course, especially after immigration, and how they influence cardiovascular disease risk. Further, specific programs^27^ that help shift gender stereotypes among immigrant women, and social and health policies^28^ that help integrate immigrants to high-income countries and adopt healthy lifestyles could help reduce sex differences in the cardiovascular health of immigrants. Lastly, investments in social policies that reduce early-life gender inequality could lead to better cardiovascular health among all.

## Supplementary Information

Supplementary Information.

## Data Availability

The datasets generated and/or analysed during the current study are not publicly available due to data access criteria that are available at www.ices.on.ca/DAS.
