# Supplementary material for: Gender inequality in source country modifies sex differences in stroke incidence in Canadian immigrants
Source: Sci Rep. 2022 Oct 26;12:17965. doi: 10.1038/s41598-022-22771-3 (PMC9605977; doi:10.1038/s41598-022-22771-3)
Supplement: Supplementary file 1 — Supplementary Information. [file 41598_2022_22771_MOESM1_ESM.docx]

**e-table 1.** Results of hierarchical frailty (random effects) cause specific models (overall cohort)

| Variable | exp(coefficient) | Standard error (coefficient) | P value |
| --- | --- | --- | --- |
| Age | 1.09 | 0.002 | <0.0001 |
| Female | 0.63 | 0.030 | <0.0001 |
| Proportion of time in Canada | 1.00 | 0.001 | 0.07 |
| Neighborhood-level income quartiles |  |  |  |
| 1^st^ (lowest) | 1.0 | (reference) |  |
| 2^nd^ | 0.97 | 0.033 | <0.001 |
| 3^rd^ | 0.95 | 0.038 | <0.001 |
| 4^th^ | 0.87 | 0.043 | <0.001 |
| 5^th^ (highest) | 0.75 | 0.054 | <0.001 |
| Immigration class |  |  |  |
| Economic | 1.00 | (reference) |  |
| Family | 1.16 | 0.033 | <0.001 |
| Refugee | 1.30 | 0.049 | <0.001 |
| Comorbidities |  |  |  |
| Hypertension | 1.91 | 0.030 | <0.001 |
| Diabetes | 1.91 | 0.032 | <0.001 |
| COPD | 1.31 | 0.058 | 0.03 |
| CHF | 1.71 | 0.087 | <0.001 |
| Dyslipidemia | 1.36 | 0.073 | <0.001 |
| Income | | | |
| GII | 0.86 | 0.241 | <0.001 |
| Female*GII | 1.75 | 0.179 | 0.002 |
|  |  |  |  |
| Random effects (group level = country of citizenship) | | | |
|  | Std deviation | Variance |  |
| Female | 0.11 | 0.012 |  |

**e-table 2.** Key Variables in the models, their sources, and operationalization

| **Variable** | **Definition** | **Data source and operationalization** |
| --- | --- | --- |
| *Exposures* |  |  |
| Sex | biological sex | RPBD. Female vs. male (binary) |
| Country of origin | Based on country of citizenship at the time of application, immigrants were classified to belong to one of the pre-specified world regions, whereas long-term residents were considered Canadian | IRCC |
| Gender Inequality Index | Based on data from the UNHCR | UNHCR |
| *Outcomes* |  | Binary outcomes |
| Incidence of ischemic stroke | An emergency department visit or acute care hospitalization with a diagnosis of ischemic stroke, using International Statistical Classification of Diseases and Related Health Problems (ICD)-10 codes: H34.1, I63.x, I64.x | CIHI-DAD or NACRS  Time-to-event outcomes |
| *Covariates* |  |  |
| Age | Biological age | RPDB. Continuous |
| Neighbourhood-level income | Based on self-reported income in postal code-linked data | Census and PCCF. Quintiles. |
| Comorbidities |  | Binary variables |
| Hypertension | ≥ 1 Hospitalization  OR  ≥ 2 physician claims in a two-year period  OR  1 physician claim followed by another physician claim or hospitalization within two years. | CIHI-DAD/OHIP. |
| Diabetes | ≥ 3 physician diagnostic code (250) in a one-year period. | OHIP. |
| Atrial fibrillation | 1 hospitalization **OR**  1 ED visit **OR**  4 physician claims in 1 year | CIHI-DAD/OHIP. |
| COPD | ≥1 Hospitalization for COPD  OR  ≥ 3 physician claims in a two-year period | CIHI-DAD/OHIP. |
| Hyperlipidemia | 1 hospitalization (ICD-9 272 or  ICD-10 E78 as any diagnosis, excluding suspect)  **OR**  2 physician claims (dxcode 272)  **OR**  1 physician claim followed by 1 hospitalization within 2 years | CIHI-DAD/OHIP. |
| Immigration class | Family – people who immigrated under the family reunification program of immigration  Economic – people who immigrated based on points-based system of immigration  Refugee - people who immigrated based on humanitarian grounds of immigration | IRCC |
| Proportion of life in Canada | Calculated as  time since immigration/(time since immigration + age at immigration) | Continuous variable  IRCC |

CIHI-DAD – Canadian Institutes of Health Information – Discharge Abstract Database

NACRS – National Ambulatory Care Reporting System

IRCC – Immigration, Refugees and Citizenship Canada

OHIP – Ontario Health Insurance Plan

ODB – Ontario Drug Benefit

PCCF – Postal Code Conversion File
RPDB – Registered Persons Database

**e-Figure 1.** Flow diagram

Immigrants, people arrived in Canada after 1985, aged 40-70 years and residing in Ontario, Canada on January 1, 2003 and a year prior

N = 509,257

Immigrants from countries for whom GII is available from UNHCR

N = 452,366

Immigrants from countries with at least 30 or more people

N = 452,089

Exclude n = 56,891

Exclude n = 277
